# Biochemical and nutritional overview of diet-induced metabolic syndrome models in rats: what is the best choice?

**DOI:** 10.1038/s41387-020-0127-4

**Published:** 2020-07-02

**Authors:** Eduardo Rodríguez-Correa, Imelda González-Pérez, Pedro Isauro Clavel-Pérez, Yolanda Contreras-Vargas, Karla Carvajal

**Affiliations:** Laboratorio de Nutrición Experimental, Instituto Nacional de Pediatría, Ciudad de México, México

**Keywords:** Biochemistry, Medical research

## Abstract

Metabolic syndrome (MS) is a condition that includes obesity, insulin resistance, dyslipidemias among other, abnormalities that favors type 2 Diabetes Mellitus (T2DM) and cardiovascular diseases development. Three main diet-induced metabolic syndrome models in rats exist: High carbohydrate diet (HCHD), high fat diet (HFD), and high carbohydrate-high fat diet (HCHHFD). We analyzed data from at least 35 articles per diet, from different research groups, to determine their effect on the development of the MS, aimed to aid researchers in choosing the model that better suits their research question; and also the best parameter that defines obesity, as there is no consensus to determine this condition in rats. For the HCHD we found a mild effect on body weight gain and fasting blood glucose levels (FBG), but significant increases in triglycerides, fasting insulin, insulin resistance and visceral fat accumulation. HFD had the greater increase in the parameters previously mentioned, followed by HCHHFD, which had a modest effect on FBG levels. Therefore, to study early stages of MS a HCHD is recommended, while HFD and HCHHFD better reproduce more severe stages of MS. We recommend the assessment of visceral fat accumulation as a good estimate for obesity in the rat.

## Introduction

Metabolic syndrome (MS) has been estimated to affect over a billion people worldwide^1^, and is defined as a pathological condition comprising the presence of abdominal obesity, insulin resistance (IR), hypertension, hyperlipidemia, and other metabolic abnormalities, which are considered as cardiovascular risk factors and that lead to the development of type 2 diabetes mellitus (T2DM). Nowadays, this condition represents the major cause of morbidity and mortality in developed and developing countries alike, representing a huge impact on their economies^2,3^.

MS results from an energy imbalance favoring fat accumulation on different tissues, this condition appears when unhealthy alimentation and a sedentary lifestyle are present, or when genetic mutations lead to this energy imbalance by inducing hyperphagia. The molecular alterations implicated in this condition include impaired or reduced mitochondrial oxidative capacity and dysregulated cellular redox state; altered insulin signaling, resulting in impaired glucose transport, and dysregulated lipolysis, all of which turn into altered lipid and carbohydrate (CH) metabolism^4^.

To help with the diagnosis of this condition, several international health organizations have established different criteria, which are summarized in Table 1.

**Table 1:** Diagnostic criteria for metabolic syndrome according to different organizations.

World health organization	Adult treatment panel III	International diabetes federation	American heart association	European group for the study of insulin resistance	American association of clinical endocrinology
• Required DM, IFG, IGT, or IR and at least two of the following: • WHR > 0.9 in men, >0.85 in women • TG > 150 mg/dL • BP > 140/90 mmHg • Urinary albumin excretion rate >2 mg/g • Albumin to creatinine ratio >30 mg/g	• Any three of the following: • WC > 102 cm in men, >88 cm in women • TG > 150 mg/dL • BP > 130/85 mmHg • HDL < 40 mg/dL • FBG > 110 mg/dL	• Required WC by the cutoffs* and at least two of the following: • TG > 150 mg/dL • BP > 130/85 mmHg • HDL < 40 mg/dL in men, <50 in women • FBG > 100 mg/dL	• Three or more of the following: • WC > 40 inches in men, and < 35 inches in women • TG > 150 mg/dL • HDL < 40 mg/dL in men and <50 mg/Dl in women • SBP ≥ 130 / 85 mmHg • FBG > 100 mg/dL	• HFI or IR and two of the following: • WC ≥ 94 cm in men, and ≥80 cm in women • TG > 2 mM • HDL < 1 mg/dL • BP ≥ 140/90 mmHg • FBG ≥ 6.1 mM	• IGT, and two of the following: • TG > 150 mg/dL • HDL < 40 mg/dL in men, and < 50 mg/dL in women • BP > 130/85 mmHg • FBG > 110 mg/dL

*DM* diabetes mellitus, *IFG* impaired fasting glucose, *IGT* impaired glucose tolerance, *IR* insulin resistance, *WHR* waist to hip ratio, *TG* triglyceride, *SBP* systolic blood pressure, *DBP* diastolic blood pressure, *FBG* fasting blood glucose, *BP* blood pressure, *HDL* high density lipoproteins, *WC* waist circumference, *HFI* high fasting insulin

*ethnicity specific values can be consulted at: www.idf.org.

It is important to note that the metabolic parameters most commonly used to determine the presence of MS among these organizations are: measurement of waist circumference, serum triglycerides (TG), and fast blood glucose (FBG) levels. Also, all of them consider that at least three parameters must be altered to define the presence of MS (Table 1).

Cellular and biochemical mechanisms implicated in the MS are highly vast and complex, and existing treatments are not fully effective, this is why animal models that mimic this disease and its metabolic and biochemical complications have been designed; they also are reproducible, efficient and accessible. Diet-induced metabolic syndrome models (DIMSM) are the most commonly used to study the MS, because of their simplicity and low cost^5^, but models consisting on pharmacological induction, spontaneous mutation, genetic manipulation, or surgical procedures also exist^6^.

In this narrative review, we thoroughly analyze existing literature using DIMSM and the different outcomes depending on the type of diet, diet duration, animal strain, animal age, and metabolic and somatometric measurements, in order to find a better consensus when trying to replicate and compare studies from different research groups.

## Metabolic syndrome models

MS is considered a multifactorial disease with environmental and genetic components, whereby genetic and dietary animal models exist; in general, genetic models are related to the mutation of the leptin receptor which induces hyperphagia, which leads to an energy imbalance that favors obesity, IR, dyslipidemias, glucose intolerance and eventually T2DM, these models are closely related to the genetic components of MS and have been reviewed elsewhere^5,6^. On the other hand, DIMSM intend to mimic unhealthy food habits and sedentarism, that has been on the rise since the past century^7^, along with an increase in fat and CH consumption due to processed food and easier availability of soft drinks and fast-food^8^ DIMSM mean to simulate this increase in CH and fat consumption, and they allow us to evaluate the effect that the excessive consumption of these two macronutrients have on metabolism and energy balance.

Most of the DIMSM are developed in rodents, as they provide benefits for studying these abnormalities: rodents present these abnormalities in few weeks, as compared with humans that can take years, also they can be early monitored during the development of the MS, and the different organs can be studied individually, as well, serum concentrations of different MS markers can be easily determined. One disadvantage when using these models is that obesity definitions have been determined for human populations, making hard to determine if these animals are obese. Even when a wide variety of DIMSM exists there is little consensus regarding the conditions needed to induce one particular alteration related to the MS, and also within the characteristics and compositions of the diets per se, making difficult to choose the appropriate model to assess a precise research question, making data from these models difficult to compare and reproduce. Also, there is not an accepted obesity definition on rodents; as for humans the World Health Organization defines obesity as abnormal or excessive fat accumulation that correlates with negative health effects. The Body Mass Index (BMI), which is determined by a person’s weight (in kilograms) divided by the square of his or her height (in meters) is the recommended parameter used to determine obesity in humans, but in rodents it is hard to determine the presence of obesity, as there is not an accurate measurement specifically determined to asses obesity in these animals. Indeed, depending on the research group, different parameters are taken into account, such as weight gain, visceral fat accumulation, dyslipidemias, etc.

Some other limitations of DIMSM include the incapacity to fully recapitulate the human pathology, as in humans the development of the disease can take years, while in rodents the observed MS develops faster and also, differences in metabolism and physiology make the disease differ between animal species and humans. These differences should be taken into account when interpreting the results obtained from a DIMSM.

Therefore, the aim of this review is to provide an analysis of existing data from DIMSM, offering a broader view of the critical points to be considered (such as: starting age, treatment duration, type of diet, etc.) when choosing the most appropriate model to answer the researcher’s question. Also, this review will compare the parameters used to evaluate obesity within each DIMSM to help resolve the best indicators of this condition.

To accomplish this objective, we first categorized the different types of diets as it follows: High Carbohydrate (HCHD), High Fat (HFD), and High Carbohydrate-High fat (HCH-HFD), and we analyzed the data from at least 35 different research groups that work with each, in order to compare the effect that each type of diet has on the development of MS. For the paper to be included in our analysis it must have: used male rats, reported some of the following MS parameters: Body weight gain (BWG), visceral fat accumulation, fasting blood glucose (FBG), fasting insulin (FI), fasting serum/plasma triglycerides levels (TG) and IR (measured by glucose tolerance tests or by the homeostatic model assessment, HOMA), and also detailed the experimental diet composition. For each paper we determined the relative change between the diet-treated and control animals of the parameters previously mentioned and this value was used to compare the severity that each diet induced over the development of the MS. We also compared the effect of diet duration and composition on the change of these parameters.

Papers related to HCHD correspond to references^9–42^, those articles related to HFD comprise references from refs. ^43–76^, and those for HCHHFD from refs. ^77–115^. Data analyzed from every paper is summarized in the supplementary Table S1.

From the data analyzed we found that DIMSM mainly differ from diet composition, duration, caloric intake, starting age of treatment, gender (usually males are selected because females tend to be resistant to diet-induced obesity), and strain, all of which are summarized on Table 2.

**Table 2:** General characteristics of DIMSM analyzed.

Type of diet	Strains	Age at the start of treatment	Variants	Administration	Percentage of carbohydrate/fat	Duration	References
High carbohydrate diet	Wistar, Sprague-Dawley	Range from 4 to 16 weeks	Isocaloric	Starch from pellet is replaced with either fructose or sucrose	Around 60%	2 to 40 weeks	^9–42^
			Hypercaloric	Either fructose or sucrose is added to drinking water	From 10% to 30%		
High fat diet		Range from 3 to 8 weeks	Low fat diet	Different sources of fat are added to the pellet	From 10% to 30%	2.5 to 25 weeks	^43–76^
			High fat diet		From 30% to 50%		
			Very high fat diet		Over 50%		
High fat-high carbohydrate diet		Range from 3 to 12 weeks	High fat-low carbs diet (HFLCHD)	Fat and CH can be added to the pellet, the drinking water or food is given to the animals directly	From 30% to 65%	4 to 20 weeks	^77–115^
			High carbs-low fat diet (LFHCHD)		From 60% to 80% in addition to standard diet		

## High carbohydrate diets

We consider an HCHD a diet in which the CH content has been increased either by modifying the CH content of the rodent chow diet, or by adding some CH to the drinking water. The first work in which an HCHD was used to induce the MS in the rat was published by Reaven’s research group in 1979^27^ and it consisted in an isocaloric high fructose diet; the content of starch in the pellet was replaced with fructose and animals were euthanized after 4 weeks of treatment. The animals reached high levels of TG, insulin, free fatty acids (FFA) and there was no change on the animal’s weight when compared with the controls. This same model led Reaven to propose the first definition of MS, which he called the “X syndrome” and that it is a precedent for the development of T2DM and several cardiovascular diseases^116^.

**Fig. 1:**
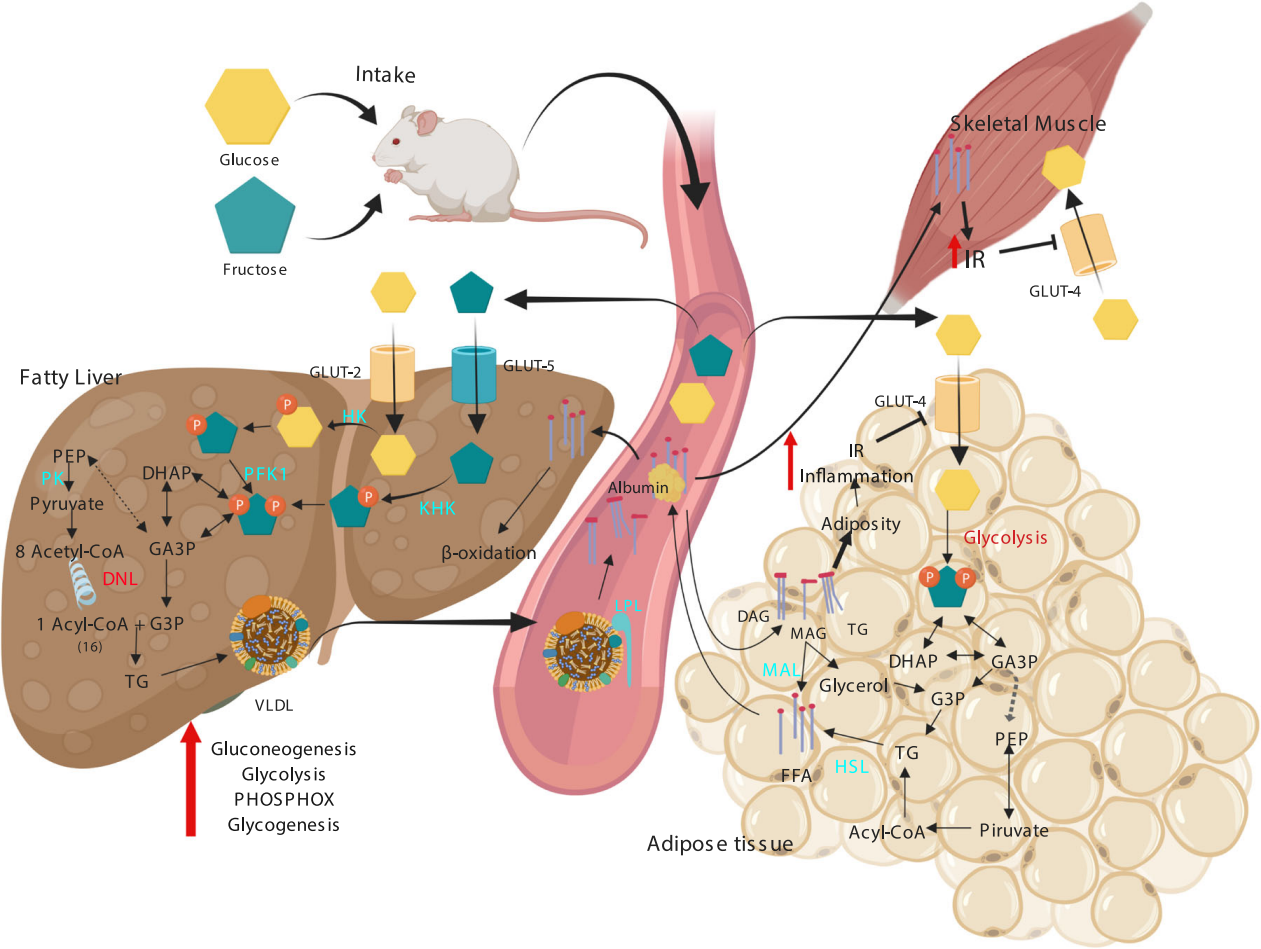
Metabolic alterations induced by a high carbohydrate diet on liver, SM and AT that contribute to the development of the MS. IR insulin resistance, HK hexokinase, KHK ketohexokinase, PFK1 phosphofructokinase 1, DHAP dihydroxyacetone phosphate, GA3P glyceraldehyde 3 phosphate, TG triglycerides, PEP phosphoenolpyruvate, PK pyruvate kinase, PHOSPHOX oxidative phosphorylation, FFA free fatty acids, HSL hormone-sensitive lipase, VLDL very light-density lipoproteins, MAG monoacylglycerol, DAG diacylglycerol, MAL monoacylglycerol lipase.

Nowadays, HCHD models can be divided in two categories: iso and hypercaloric diets, in the first one the standard CH of the commercial diet (starch, which is a complex polysaccharide) is usually replaced with simple CHs that have higher energetic availability like fructose or sucrose (which is a disaccharide composed of one unit of glucose and one unit of fructose). On the other hand, hypercaloric diets results from adding either fructose or sucrose to the drinking water; the animals decrease their solid food consumption but their caloric intake is increased when compared with animals fed the standard chow^13^. Both iso-caloric and hyper-caloric HCHD, mainly exert their effects because the simple sugar that the experimental animals consume increases the substrate availability for several metabolic pathways. Adding fructose to the diet has diverse metabolic implications which all lead to the development of metabolic abnormalities and eventually to the MS. Once ingested, fructose is taken by the liver from the blood (around 70% of all ingested)^117^ where is rapidly phosphorylated by the liver ketohexokinase (KHK, fructokinase) to generate fructose-1-phosphate (F1P), this process is not sensitive to the cellular energy status, as it occurs for the glycolytic pathway, which is regulated upstream by the inhibition of the phosphofructokinase type I (PFK-I) step of glycolysis; the F1P produced from fructose is metabolized to dihydroxyacetone phosphate (DHAP) and glyceraldehyde 3-phosphate (G3P), which in turn feed the hexose and triose phosphate pools, increasing the carbon source for the central carbon metabolic pathways including glycogenesis, lipogenesis, glycolysis, gluconeogenesis and citric acid cycle. The other metabolic pathway actively augmented by fructose is the hepatic lipogenesis, since it provides intermediates for lipid synthesis and fatty acid oxidation inhibition, which in turn increases the production and secretion of very light density lipoproteins (VLDL). These VLDL are rich in TG that can be hydrolyzed by the lipoprotein lipase (LPL) and stored by the adipose tissue, leading to obesity (for more detailed information on fructose metabolism the review of Hannou SA, et al. 2018 is recommended)^118^. The adipose hypertrophy increases inflammation, which in turn favors IR in the fat cell, this reduces the lipolysis inhibition mediated by insulin resulting in an increase of FFA liberated to the blood torrent, FFA are then taken up by other organs like the liver and skeletal muscle (SM), this FFA are stored by these tissues and induce metabolic abnormalities that aggravates peripheral IR (Fig. 1).

**Fig. 2:**
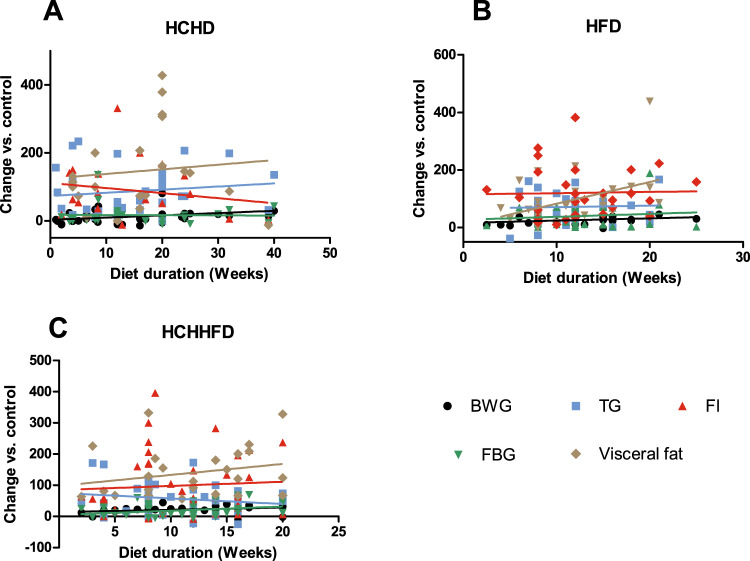
Analysis of the diets effect on metabolic parameters. Dots represent the data acquired from each analyzed report. BWG body weight gain, TG triglycerides, FI fasting insulin, FBG fasting blood glucose.

In this study, we analyzed 22 papers reporting hypercaloric and 15 isocaloric diets and we compared the effect of the diets on the parameters previously mentioned. When using this kind of diets, we found that the duration of these diets varied from 2 to 40 weeks and the age at the start of treatment ranged from 4 to 16 weeks, also the most common used strain was the Wistar (Table 2). The main affected parameters were TG, visceral fat accumulation, IR and FI; regarding BWG and FBG no effect or little significant increase were found (Table 3) (Fig. 2).

**Table 3:** MS parameters reported as increased by each type of diet.

Diet	Strain	BWG	TG	FI	FBG	Fat%	IR
HCHD	80% Wistar, 20% Sprague–Dawley	+	+++	+++	+	+++	++++
HFD	60% Wistar, 40% Sprague–Dawley	+++	+++	+++	+++	++++	++++
HCH-HFD	79% Wistar, 21% Sprague–Dawley	+++	+++	++	++	++++	+++

The symbols represent the percentage of articles that reported a statistically significant increase on the parameter as it follows: “+” = 0–40%, “++” = 40–70%, “+++” = 70–90%, and “++++” = 90–100%.

*BWG* body weight gain, *TG* triglycerides, *FI* fasting insulin, *FBG* fasting blood glucose, IR insulin resistance.

Among the HCHD it exists a variability in the CH dosage, duration and administration, all of which can create confusion at the moment of choosing the appropriate diet for a particular research study. From the data here analyzed, we found that most of the studies used the hypercaloric diet and that this diet had a stronger effect on BWG than the isocaloric one. Hypercaloric diets exerted a mild effect on the BWG of about 18%, while in the isocaloric diets this increase was almost inexistent (only about 2%) (data not shown). Large number of articles using hypercaloric diet reported a significant increase in BW when compared with the control groups (47%) and only 29% of the isocaloric diets reported the same increase. Regarding the rest of parameters, no significant difference between the two HCHD was found, but there was a tendency for every parameter to be higher in the hypercaloric diet (Data not shown). This indicates that hypercaloric diet is the predominant choice when inducing the MS, due its lower cost and easy preparation, although isocaloric diets allow the introduction of a different nutrient by modifying the standard chow composition. These diets are commonly used to evaluate the effect of different nutrients and substances on preventing or reverting the development of the MS^10,17,23,106,119,120^.

Since hypercaloric diets induce a greater effect on MS features, probably because of their higher energetic availability of this type of diet, it is noteworthy to state the effect that different sucrose concentrations have on the development of the MS. In this sense, Acosta-Cota SJ et al. 2019 evaluated the effect of adding 30, 40, and 50% of sucrose to the animals drinking water and found that after 20 weeks of treatment the animals receiving 40% and 50% sucrose had significant increase in BWG, but not those receiving the 30% dose, which is the concentration most commonly used in this type of diets. Also, they found that all the doses induced IR when determined by an oral glucose tolerant test after 20 weeks, and that the degree of hepatic damage increased with diet duration and sucrose dose^35^.

**Fig. 3:**
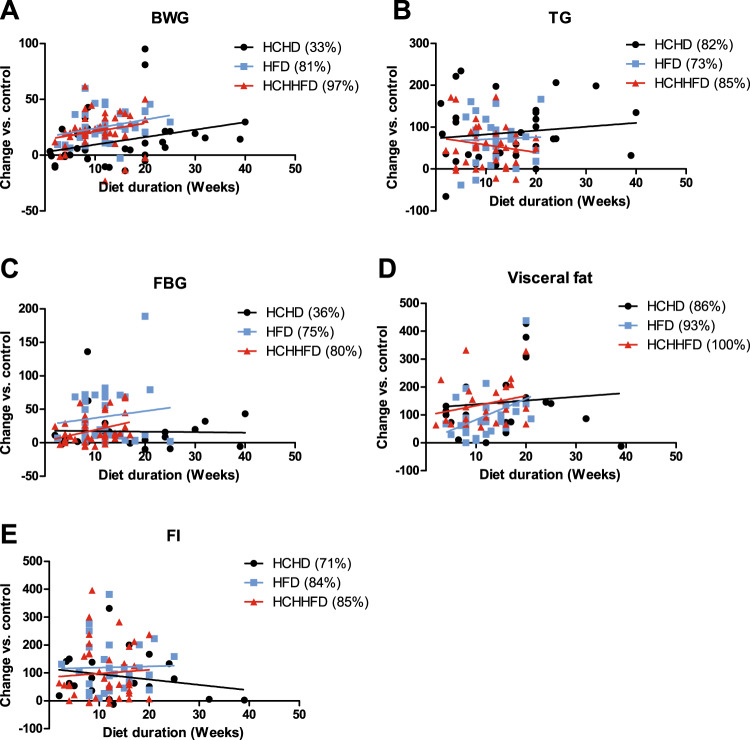
Comparison between diets of the effect on the MS parameters. Dots represent the data acquired from analyzed reports. BWG body weight gain, TG triglycerides, FI fasting insulin, FBG fasting blood glucose. The numbers between brackets represent the percentage of articles that reported a statistically significant increase of the parameter.

Regarding BWG, we found that 33% of the research groups that used HCHD reported a statistical significant increase when compared with the control groups in both types of diets (Fig. 3a), however this increase was only about 12% (Fig. 2a) so that, these diets have a mild effect on this parameter, especially during the first weeks of treatment, as the effect becomes more evident around the 25th week of treatment (Fig. 3a), this is why when trying to achieve an increase in BW by using these diets we recommend giving the diet for more than 20 weeks.

The FBG levels were only reported significantly increased by 36% of the research groups (Fig. 3c), and the mean value of this increase was around 17% (Fig. 2a), all of this indicates that the animals subject to HCHD do not develop a condition similar to T2DM. Nonetheless, the FI levels of these animals where reported as significantly increased by 71% of the groups (Fig. 3d), and it does not seem to correlate with the duration of the diet (Fig. 3d, Fig. 2a). This is supported by the findings from several groups that reported an increase of around 60% of the FI levels regardless the duration of HCHD^11,12,26,121^.

Concerning IR, we found that 95% of the groups reported a condition of IR (IR was determined by glucose and insulin tolerant tests, HOMA indexes, and glucose uptake rate in some tissues) (Supplementary Table 1). All of this indicates that animals subjected to a HCHD are developing peripheral insulin resistance, which in turn may lead to pancreatic β-cells to overproduce insulin in order to compensate IR; this condition is known to be the previous step for T2DM onset, as the pancreas fails shortly after that, resulting in sustained higher blood levels of glucose^122^.

Considering the effect of HCHD on TG serum levels, we found that 82% of the research groups found a significant statistically increase of this parameter when compared with the control group receiving standard diet (Fig. 3b), most of the authors reported that this parameter increases within the first weeks of the diet and remains constant during longer periods on HCHD (Fig. 2a). Interestingly, even when the effect of the HCHD on BWG was not considerable, 86% of the groups reported a significant increase on visceral fat (Fig. 3e). The increase on visceral fat induced by HCHDs reaches around 150% over the control animals, and it does not seem to be related to the diet duration (Fig. 3e). Furthermore, it has been reported that animals drinking 30% sucrose water during 26 weeks increased their global adipose tissue by 24%, and decreased their skeletal muscle mass by 18%^122^, this could explain why we found that the effect of HCHD was greater on visceral fat accumulation than on BWG, and it also suggests that measuring visceral fat accumulation would be a better indicator of obesity than BWG in these animal models.

All these findings indicate that HCHD models induce an impairment on lipids metabolism and IR, but are not effective on inducing a significant weight gain or increased basal glucose levels, thus these models resemble and early stage of MS, a condition that precede T2DM.

Taking all of these into account, in order to get an effect on obesity, we recommend the use of HCHD at a dose of 30% in the drinking water, as it is easier to dissolve and achieves changes in the majority of the MS parameters. However if an increase in BWG is desired, more than 25 weeks of treatment should be used, or higher sucrose concentrations. We also recommend that other obesity parameters are determined (particularly visceral fat deposition) as BWG is not always increased. It is also important to remark that these models are not the best ones to study T2DM, but are suitable to study the previous stages that lead to this disease, when IR predominates. In conclusion, these types of diets have a high utility as they induce alterations in the majority of the MS parameters, they also have low costs, and they are usually easy to produce, since sucrose is a common CH used as a commercial sweetener that is easy to obtain.

### High fat diets

We consider a high fat diet (HFD) that in which fat percentage is over 10%, usually by exchanging the CH content for fat, which makes this type of diets hypercaloric. In 1955 Mickelsen, Takashi and Craig worked with one of the first HFD, in this research they used two strains of rats: Sprague-Dawley adult males rats and Osborne–Mendel weanling males rats. The diet was composed of over 65% of hydrogenated fat, and was administered for more than 40 weeks. The animals stopped gaining weight at week 40, and after that they started to lose weight. Surprisingly Osborne–Mendel rats fed with the HFD reached weights ranging from 900 g to 1300 g, proving that a HFD could induce a significant increase in BW. On the other hand, Wistar rats did not increased their body weight gain when compared with their respective controls^123^.

The influence of HFD on energy metabolism is dependent on the fat type added to the diet, for the HFD the most commonly type of added fat is composed of equal proportions of saturated fatty acids and monounsaturated fatty acids, which are usually contained within lard. This type of fatty acids has been reported to lead to the most pronounced manifestations of obesity and IR, when compared with diets rich in the other types of FA, such as coconut oil or fish oil^54^, while oils rich in polyunsaturated ω-3 fatty acids have beneficial effects on body composition and insulin sensitivity^124^. Considering this, the data analyzed in this review mostly included articles using HFD rich in saturated fatty acids (usually diets containing lard).

**Fig. 4:**
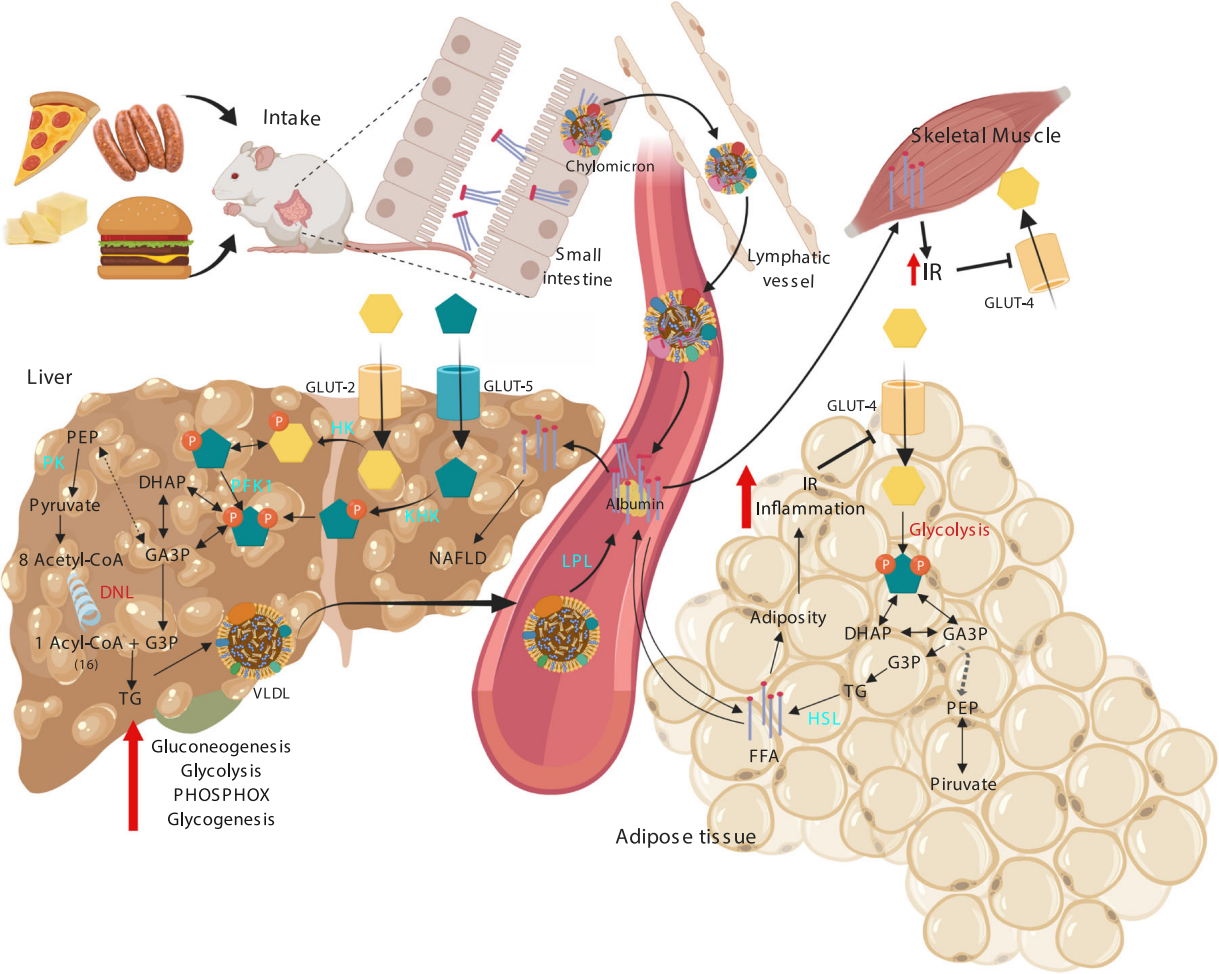
Metabolic alterations induced by a high fat diet on Liver, SM, and AT that contribute to the development of the MS. IR insulin resistance, HK hexokinase, KHK ketohexokinase, PFK1 phosphofructokinase 1, DHAP dihydroxyacetone phosphate, GA3P Glyceraldehyde 3 phosphate, TG triglycerides, PEP phosphoenolpyruvate, PK pyruvate kinase, PHOSPHOX oxidative phosphorylation, FFA free fatty acids, HSL hormone-sensitive lipase, VLDL very light-density lipoproteins.

Once ingested, TG from HFD are broken down into FFA by pancreatic enzymes and bile salts and are absorbed by the enteric cells of the small intestine, there they are then packed with cholesterol into phospholipid vesicles called chylomicrons that travel through the lymphatic system and then reach the circulatory system, increasing the serum concentrations of these lipoproteins. By means of lipoprotein lipase (LPL) these different fats are hydrolyzed into FFA that are taken by the adipose tissue cells, re-esterified into TG and stored as lipid droplets leading to obesity (Fig. 2). At the same time, the liver can take the FFA and accumulate them in fat droplets which lead to non-alcoholic fatty liver disease (NAFLD) and also induce hepatic lipogenesis, increasing the serum FFA even further, and promoting the formation of cholesterol. FFA are substrate for the formation of Acetyl CoA that feeds the Krebs cycle and the respiratory chain and consequently, altering the cellular redox state^6,99^ (See Fig. 4). High levels of serum FFA induce IR in adipose tissue and skeletal muscle which in turn, fail to regulate serum glucose and FFA levels. Briefly, these are the mechanisms by which the HFD may directly increases adiposity, along with the other abnormalities of the MS^125^.

According to the content of fat relative to chow weight, there is a categorization for this diet: low fat diet (LFD), high fat diet (HFD) and very high fat diet (VHFD). Usually standard diets contain 5 to 10% of fat, while LFD ranges from 10 to 30%, HFD from 30 to 50% and VHFD over 50% of the total fat relative to chow weight^126^ (Table 2). In addition, the duration of the diets span from 2.5 to 25 weeks and the most common used strains were Wistar and Sprague–Dawley (Table 2).

From the papers analyzed in this review, we found that HFD that ranged from 30 to 50% of fat were the most commonly used, we analyzed 6 papers reporting LFD, 22 using HFD and 10 VHFD, we compared the effect on the MS parameters among the three types of HFD, finding that the three types of diet induced the same increases in TG, FI and visceral fat accumulation, however, VHFD had a lower effect on BWG and FBG, this could be due to the reduced CH content of these diets that makes them similar to the ketogenic diets, which are able to reduce the BW of the animals^127^.

When analyzing all the data from the revised reports we found that 81% of all the groups using the HFD reported a significant increase in BW and this increase seems to correlate with the diet duration (Table 3, Fig. 2b). Concerning TG levels, 73% of the research groups reported a significant increase in this parameter when compared with the standard diet groups. HFD induces an increase around 80% on TG with no apparent relation to the diet duration (Fig. 3b), suggesting that the dyslipidemia induced by these diets starts early. In a similar way, FI levels had a two-fold increase when compared to the control groups, as reported by 84% of the analyzed studies. This effect does not seem to correlate with the duration of the diet (Fig. 3d), suggesting that peripheral IR also develops early when consuming a HFD, as reported by Ciapaite et al., 2011^71^. In the case of FBG levels, 75% of the groups reported a significant increase on this parameter, HFD seem to induce the greatest and fastest increase in FBG, even some studies reported a significant increase at only 8 weeks of fat consumption^53,56,57^ (Fig. 3c). Regarding visceral fat accumulation the percentage of visceral fat was reported significantly increased by 93% of the groups, and it seems to be proportional to the diet duration, thus the effect of the diet on visceral fat becomes more apparent after 10 weeks, with a strong rise from this time; this effect is different from the HCHD in which the accumulation of visceral fat appears to increase quickly and stays constant during longer periods of CH consumption (Fig. 3e).

As already mentioned, when using a HFD it is important to consider the ketogenic effect of VHFD, which can lead to lose body weight, and confers metabolic benefits, like improved insulin sensitivity, improved glucose tolerance and lower TG levels^128,129^, being counterproductive to the development of MS. Therefore, in order to obtain the features of MS, we recommend the use of a 30% to 50% HFD, as those are the most commonly used and commercially available, allowing data comparison among other research groups. Contrary to HCHD, we can say that HFD induces the development of a condition similar to T2DM within the first 10 weeks of treatment, as these diets were the most effective on altering the FBG. We conclude that these types of diets are recommended for studying T2DM and its related abnormalities, and at least 10 weeks of treatment are recommended (Fig. 3c).

Another important point to consider when working with these diets is that in many cases rats are fed a HFD using standard chow diet with added fat, it may be considered that t there could be nutritional inadequacies, the addition of too much fat can dilute other nutrients like soluble vitamins or minerals^126^. It is recommendable that every research group adapts the caloric intake when adding fat to a standard chow diet, but this arise another problem: the lack of standardization of the diet composition between different research groups. When fat is added manually, it becomes difficult to ensure the exact quantity of kilocalories that the rats are consuming. Also, it could render the food less palatable, this could interfere with the amount of food consumed by rats. These problems can be attenuated by using a manufactured laboratory diet provided by a commercial provider, nonetheless this is more expensive and somewhat difficult to acquire in several countries.

### High carbohydrate-high fat diet

In 1976, the scientist Sclafani was interested in the syndrome of hyperphagia/obesity that resulted from lesions of the ventromedial hypothalamus (satiety center), he observed that the same effects could be obtained without surgery in rats by a HCH-HFD dietary intervention, which consisted in 33% fat standard chow plus a variety of freely accessible, highly palatable commercial foods such as: sweetened condensed milk, chocolate chips, cookies, salami, cheese, banana, marshmallows, milk chocolate and peanut butter, given for a period of two months, and he named it the cafeteria diet^130^. The animals fed with the HCH-HFD gained 53% more weight than the control group receiving standard chow, a value even higher than that reported by Mickelsen et al. 1955 and Peckham et al., 1962^123,131^ who used a HFD model in which the treatment lasted 2 to 4 times longer^41^.

Since then, this diet has been widely used and it represents a robust model capable to induce most of the metabolic disorders that occur in MS (obesity, dyslipidemia, hyperinsulinemia, glucose intolerance, IR, hyperglycemia, and inflammation), it also closely reflects the style of nutrition of the western population, where the consumption of processed, pleasant-tasting and highly energetic foods predominate^5,84,86,110^. The unhealthy effects produced by the HCHHFD diet are well characterized; Hazarika et al. have reported structural and physiological abnormalities in organs such as the heart, liver, kidney, pancreas, adipose tissue, lung, spleen, and small intestine at week 12 and 16 of the diet^99^.

All these effects are interconnected and can be explained at a biochemical level, the metabolism of carbohydrates and fats that derive from the cafeteria diet enhance the rise of fructose, glucose, and lipids that circulate in the blood and enter the tissues, disturbing the metabolic pathways previously simplified in Fig. 1 and Fig. 2. Particularly in animals fed with HCH-HFD, these routes are over-stimulated, especially the glycerolneogenesis pathway, the main supplier of glycerol-3-P for lipid synthesis in the liver tissue. Also, the gluconeogenic capacity increases and not only due to hepatic insulin resistance, but also it has been proposed that glycerol derived from TG breakdown may directly feeds this pathway^132^. In addition, the activity of the fructose-1,6-biphosphatase is increased, as well as the glucagon/insulin ratio. The higher rates of gluconeogenesis probably contribute to hyperglycemia and a greater synthesis and deposition of hepatic glycogen under a hyperinsulinemic condition, the latter produced by a hyperactivity of the β-pancreatic cells produced as a compensatory mechanism to IR^6,99,133^.

The main characteristic that makes cafeteria diet different from the HFD or from the HCHD is the use of highly palatable processed foods such as biscuits, wafers, condensed milk, sausages and soft drinks, which unlike the food in pellet, can induce a mechanism of neuronal adaptation where appetite regulation is altered, inducing pleasure and activating the reward system. These resemble the behavior that promotes the use of drugs, since it increases the motivation to consume highly energetic food contributing rapidly to the development of obesity^134,135^. The HCH-HFD diet has shown a greater increase in body weight and adiposity, in comparison to the use of modified diet pellets^95^.

On the other hand, we observed that 60% of the analyzed articles, reported a reduction of the protein content replaced by fat, which confers a disadvantage to this type of model, because low protein intake may lead to different metabolic abnormalities, for example, it has been reported that a low protein diet induces the loss of body weight and brown and white adipose tissue, which is clearly not the objective of this diet, and generates a significant impact on the BWG, visceral fat and insulin values^81–83,85,97,102^^,136^. When using this type of diets, it is recommended that the protein content remains as stable as possible to avoid such alterations.

According to the articles analyzed, there are two main types of HCH-HFD diet depending on the macronutrient that predominates in the mixture forming the pellet or the processed food offered to the animals, it can be high in fat (30–65%) and low in carbohydrates or high in carbohydrates (60–80%) (Table 2).^137^ The majority of the authors choose a diet with a higher proportion of fat (85% of the those analyzed) because the cafeteria diet usually includes a high content of polyunsaturated fatty acids and a low proportion of protein, that leads to an increase in thermogenic activity and rapidly induces hyperphagia increasing food and caloric intake^138^. The rest of the authors chose to increase the carbohydrate content and, in exceptional cases, increases both components.

A disadvantage that shares with the other diets is the high variability in the duration of the diet (from 2 to 20 weeks), as well as its composition. The age at the start of diet is another factor to consider, which is even more variable (from 3 to 60 weeks) than the HFD or HCHD and depends on what the researcher intends to study (Table 2). Very few authors initiate the diet in weanling rats, however there is evidence that animals feeding with this type of diet at early stages of life, can enhance the pathological effects during the adult age in the rats^44^. It was also found that the Wistar strain was the most commonly used by the researchers (Table 2).

All of the articles analyzed reported a statistically significant increase on visceral fat (Table 3). In the case of BWG, it was found that 89% of the articles found a significant increase compared with their respective control group and, although there was no association with the duration of treatment, it is not advisable to use periods of time shorter than 5 weeks if you want to observe an effect on this parameter (Fig. 3a).

When assessing obesity, all the groups reported an increase in the proportion of visceral fat, which ranges from 50% to 300% from the control animals (Fig. 2c), which indicates that the cafeteria diet may be the most suitable to evaluate effects on adipose tissue, due to its considerable effectiveness in increasing this parameter, as well as BWG. As previously noted for the HCHD and the HFD (Fig. 3a), the visceral fat accumulation is a good parameter to estimate obesity, and it is also increased on this type of diet, but to a higher extent. In addition, it has been described that animals fed with the HCH-HFD diet have greater infiltration of macrophages in white and brown adipose tissue, accompanied by body weight increase and severe adiposity, when compared with a HFD diet that contained a similar proportion of fat (40–50%), but a lower caloric content^111,112^ therefore suggesting that rats fed with a HCH-HFD increase their food consumption because of the greater palatability, and not the diet composition per se.

In the case of serum TG levels, 81% of the articles reported a significant increase with respect to the controls (Table 3). Increases over 40% can be observed at 2 or 20 weeks of diet, so this parameter is totally independent of the diet duration (Fig. 2c). 59% of the reports included in this review revealed a significant increase in FBG, and it seems to be associated with the duration of the diet (Table 3); FBG levels increased up to 30% from the control before the 7th week and after that time it raises up to 50–70% (Fig. 3c). The fact that FBG significantly increases from the second week of treatment may be due to the fact that the high content of fat of the diet accelerates β-cell dysfunction, avoiding the glucose compensatory mechanisms that occur during the early stages of IR, as it happens in the HCH-HFD. The fact that this parameter was reported to be increased by only 59% of the articles may be related to the low reproducibility of the diets, due to the high variability in diet composition.

Concerning FI levels, 82% of the analyzed articles reported hyperinsulinemia in the animals fed with this diet, increases over 50% from the control can be observed within the two first weeks of treatment, however with a period of 8 weeks of treatment, increases over 100% can be obtained (Fig. 3d). Likewise, in parallel with the development of hyperinsulinemia, 78% of the research groups using the cafeteria diet reported IR by analysis of HOMA-IR and glucose tolerance tests (Table 3). Paschen et al. 2019 conducted a study where they evaluated the effect of HCH, HFD and HCH-HFD at 4 and 8 weeks of treatment on the development of β-cellular IR, showing that only the animals under HCH-HFD were able to develop it^133^. They also found a concomitant loss of functional mass of β cells, so they suggested that HCHFD is a good model to evaluate the progression stages from MS to T2DM^133^.

It can be concluded that the HCH-HFD is a suitable and reliable intervention to effectively induce most of the metabolic disorders that occur in the MS. Besides, it mimics western human diet by providing high fat-processed foods and refined CH but low in protein, vitamins and minerals. Nevertheless, the major limitation of this diet is that its nutritional composition is heterogeneous and it is complicated to calculate the daily intake, making difficult to compare results from independent research teams, however the most common used cafeteria diet is composed of 60% fat with 30% of carbohydrates and 10% of protein, with duration of 8 weeks or more.

### Defining obesity in the rat model

From all the studies included in this review, it can be rescued that one parameter that is constantly affected is visceral fat, no matter the chosen diet, and the period is given for, fat depots increase significantly. Indeed, obesity has been defined by the World Health Organization as the abnormal fat deposition that causes unhealthy profiles in an individual, since BW or BMI does not accurately reflect adiposity in the rat, these parameters are poor indexes of obesity in these animals, and also fail to describe the obesity levels in some human populations. Moreover, recently it has been demonstrated that in humans, the golden standard to determine obesity is the determination of whole fat composition by Dual-energy X-ray absorptiometry (DEXA or DXA), as it correlates better with the obesity level^139^. This outlines how visceral fat better defines obesity, even in humans. Due to the poor availability of biological imaging procedures for small animals, such as DEXA or computerized axial tomography, to evaluate fat composition, visceral fat content, measured by weighing major fat pads, such as retroperitoneal, inguinal or omental depots, emerges as an accurate tool to assess the effect of nutritional interventions on obesity development. In this sense, it has been shown that any of these fat depots are affected at the same extent under obesity-induced interventions^123^. Moreover, most of the studies analyzed, regardless the type of diet, reported metabolic consequences such as hyperlipidemia, hyperglycemia, inflammation and IR, all of which are associated to obesity, and are referred as diet-induced obesity, even though there is not always a significant increase in body weight.

### Comparison among the diets, which one should I use?

Different DIMSM offer different alternatives that allow the researcher to choose based on economic and methodological bases, the best model to use. Here we summarize the different characteristics, advantages and disadvantages and the mainly outcomes that each dietary intervention provide.

In this sense HCHD are recommended to study the early stages of MS that precedes the onset of T2DM, since in these models there is a mild effect on the FBG. On the other side HFD and HCHHFD have larger effects on this parameter. We also found that HFD and HCH-HFD induced the MS faster than the HCHD (Fig. 3), so when wanting to have a faster development of the MS these diets are recommended, these diets are also the recommended when trying to simulate a condition close to T2DM, as they increase the FBG in most of the cases (Fig. 3c).

Regarding the determination of obesity on the rat, we found that high carbohydrate consumption seems to have a smaller effect on BWG than the diets that are rich in fat, but the effect on visceral fat accumulation was similar in all the diets (Fig. 3e), this could be explained by the previously reported reduction of skeletal muscle mass due reduced food consumption which in turn results in a lower protein consumption when eating a HCHD^122^,therefore we suggest that visceral fat accumulation is a better parameter to determine obesity, as it is increased in a similar proportion in all the diets (Fig. 3e).

FI behaves in a similar way among the diets which indicates that peripheral tissues become IR when treated with any of these type of diets, but for the FBG levels we found that HCHD is less effective in modifying this parameter (Fig. 3c, d), which indicates that HCHD has a lower effect on pancreatic β-cell dysfunction, which allows the compensatory effects for IR to last longer.

When using a HFD we recommend a fat proportion between 30% and 50% as higher values could induce metabolic abnormalities unrelated to the MS, and it is also recommended, If possible, acquiring and using a commercial HFD chow to make results comparable with other studies.

HCH-HFD can induce most of the abnormalities related to the MS but there is little consensus between the diet composition, making difficult the comparison of data among different groups and the design of the right diet. As this heterogeneity exists, we strongly advise that studies using HCH-HFD should meticulously report the diet composition and the caloric intake of the animals.

As a recommendation, researchers not only should be careful when choosing and reporting the nutritional components of the chosen diet to induce MS, but also with the control diet. It has been shown that some components of the control diet (such as natural antioxidants) can attenuate the diet effect on insulin resistance and weight gain^140^.

Due to its agricultural byproducts source, and its closed formula, election of a standard chow diet could bring variability and a difficult reproducibility^141^ as the diet composition may vary between batches^142^. Altogether, we recommend using a purified control diet, because purified ingredients are used as source of the macronutrient that distinguishes the type of diet and provide an “open source” formula, making it easier when comparing across studies from different research groups^141^

In conclusion, multiple DIMSM exist and none of them fully resemble the human pathology, as animals differ in their metabolism and physiology, also each type of diet exerts different metabolic abnormalities at different times of consumption. Nevertheless, these models are very useful to study the MS and their use has provided valuable results that should lead Health authorities to encourage and promote the consumption of low calorie, low fat/sugar diets and increasing daily physical activity for people so, hopefully one day these DIMSM are no longer needed, since prevention is the best cure for MS and more severe associated comorbidities.

## Supplementary information

Table S1
