# Supplementary material for: Biochemical and nutritional overview of diet-induced metabolic syndrome models in rats: what is the best choice?
Source: Nutr Diabetes. 2020 Jul 2;10:24. doi: 10.1038/s41387-020-0127-4 (PMC7331639; doi:10.1038/s41387-020-0127-4)
Supplement: Supplementary file 1 — Table S1 [file 41387_2020_127_MOESM1_ESM.pdf]

Table S1. Raw data from analyzed papers and source references

Strain	Type of diet	% main nutrient	Starting age (weeks) or weight (g) of treatment	Diet duration (weeks)	Final Weight (g)		Change vs Control	Triglycerids		Change vs Control	Insulin		Change vs Control	Blood glucose		Change vs Control	Fat accumulation (g)		Change vs Control	Insulin resistance	Research group	Reference Number
					Control	Experimental		Control	Experimental		Control	Experimental		Control	Experimental		Control	Experimental				
Wistar-Han	HCHD^{I}	65 starch or fructose + 10 oligofrutose	N/A	4	330	318	-3.6	3.73 mg/dL	2.49 mg/dL	-33.2	712 µU/mL	533 µU/mL	-25.1	9.6 mmol/L	9.7 mmol/L	1	17.1	34.2	99.9	N/A	Guzmán-Gerónimo RI , et al 2017	9
Wistar	HCHD^{H}	30 sucrose	3	12	400.7	359.2	-10.4	1.7 Mm	2.3Mm	35.3	N/A	N/A	N/A	4.9 mmol/L	5.6 mmol/L	14.3	12.8	10.5	-18.0	N/A	Guzmán-Gerónimo RI, et al, 2017	9
Wistar-Han	HCHD^{I}	60 starch or fructose	N/A	8.5	182.9	226.4	23.8	N/A	N/A	N/A	53.3 µU/mL	96.8 µU/mL	81.6	76.8 mg/dL	181.2 mg/dL	135.9	N/A	N/A	N/A	Yes	Sreeja S, et al, 2014	10
Wistar-Han	HCHD^{I}	50-60 starch or fructose	N/A	8.5	211.7	205.2	-3.1	N/A	N/A	N/A	53.4 µU/mL	58.4 µU/mL	9.4	77.9 mg/dL	84.9 mg/dL	9.0	N/A	N/A	N/A	Yes	Sreeja S, et al, 2015	10
Sprague-Dawley	HCHD^{H}	30 sucrose	N/A	5	N/A	N/A	N/A	91mg/dL	304 mg/dL	234.1	26 µU/mL	40 µU/mL	53.8	N/A	N/A	N/A	N/A	N/A	N/A	Yes	Sharabi Y, et al, 2007	11
Wistar	HCHD^{H}	30 sucrose	4	20	433	410.9	-5.1	0.79 mmol/L	1.73 mmol/L	119	7.6 µU/mL	11.5 µU/mL	51.3	N/A	N/A	N/A	13.2	34.6	162.1	N/A	El Hafidi M, et al, 2001	12
Wistar	HCHD^{H}	30 sucrose	N/A	20 a 27	428	478	11.7	153.8 mg/dL	264.5 mg/dL	72.0	N/A	N/A	N/A	N/A	N/A	N/A	N/A	N/A	N/A	N/A	Carvajal K, et al, 1999	13
Wistar	HCHD^{H}	30 sucrose	4	20	440	526	19.5	0.5 mmol/L	1.2 mmol/L	140.0	106.8 pM	285.2 pM	164.0	6.2 mM	5.6 mM	-9.7	2.9	15.3	427.6	N/A	Ruiz-Ramirez A, et al, 2010	14
Wistar	HCHD^{I}	68 starch or fructose	N/A	7 a10	544	525	-3.5	N/A	N/A	N/A	94 mg/dL	128 mg/dL	36.2	125 mg/dL	127 mg/dL	1.6	N/A	N/A	N/A	N/A	Dutta K, et al,2001	15
Sprague-Dawley	HCHD^{I}	N/A	5	2	102	91	-10.8	25 mg/dL	34 mg/dL	36.0	1.1 ng/mL	1.3 ng/mL	18.2	45 mg/dL	50 mg/dL	11.1	N/A	N/A	N/A	N/A	Koo HY, et al, 2008	16
Wistar	HCHD^{I}	55.5 -58 starch or fructose	N/A	12	465	522.5	12.4	0.78 mmol/L	2.32 mmol/L	197.4	435.3 pmol/L	457 pmol/L	5.0	6.5 mM	8.4 mM	29.2	N/A	N/A	N/A	Yes	Creus, et al,2017	17
Wistar	HCHD^{I}	62.5 starch o sucrose	N/A	32	472.1	545	15.4	0.69 mmol/L	2.06 mmol/L	198.6	70.5 mU/mL	74.7 mU/mL	6.0	6.29 mmol/L	8.31 mmol/L	32.1	8.1	15.1	86.9	N/A	Illesca G, et al, 2017	18
Wistar	HCHD^{I}	63 sucrose	N/A	6.4	234	248	6.0	N/A	N/A	N/A	N/A	N/A	N/A	N/A	N/A	N/A	N/A	N/A	N/A	N/A	Lombardo YB ,et al, 1983	19
Wistar	HCHD^{I}	81.3 sucrose	N/A	4	N/A	N/A	N/A	118.75 mg/dL	139.75 mg/dL	17.7	N/A	N/A	N/A	109.55 mg/dL	113.88 mg/dL	4.0	N/A	N/A	N/A	N/A	Toida S, et al, 1996	20
Wistar	HCHD^{I}	81.3 sucrose	N/A	12	N/A	N/A	N/A	91.5 mg/dL	142.22 mg/dL	55.4	N/A	N/A	N/A	116.12 mg/dL	141.55 mg/dL	21.9	N/A	N/A	N/A	N/A	Toida S, et al, 1996	20

Wistar	HCHD[H]	20 sucrose	N/A	24	535	650	21.5	43 mg/dL	74 mg/dL	72.1	N/A	N/A	N/A	76 mg/dL	82.5 mg/dL	8.6	5.5	13.5	145.5	N/A	Velasco M, et al, 2012	21
Wistar	HCHD[H]	20 sucrose	N/A	8	366	488	33.3	45 mg/dL	50 mg/dL	11.1	2.37 pmol/L	29.2 pmol/L	1132.1	5.2 mg/dL	5.4 mg/dL	3.8	2.0	6.0	200.0	Yes	Albarado-Ibanez A, et al, 2013	22
Wistar	HCHD[I]	25 sucrose	N/A	20	495	447.8	-9.5	69.1 mg/dL	139.1 mg/dL	101.3	N/A	N/A	N/A	5.2 mg/dL	5.4 mg/dL	3.8	N/A	N/A	N/A	N/A	Sousa, RML, et al 2018	23
Wistar	HCHD[I]	31.5 glucose	7	6	1.87	2.22*	18.7	137.24 mg/dL	184.1 mg/dL	34.1	N/A	N/A	N/A	78 mg/dL	79.4 mg/dL	-5.6	5.1	5.7	10.6	N/A	Sousa RML, et al ,2018	23
Wistar	HCHD[H]	30 sucrose	1.7	25	419.67	509.2	21.3	N/A	N/A	N/A	7.61 µU	13.62 µU	79.0	110.63 mg/dL	100.73 mg/dL	-8.9	4.1	9.8	140.7	N/A	Villegas-Romero, et al, 2018	24
Wistar	HCHD[H]	10.6 (regular cola beverage)	1.7	24	626	669	6.9	80 mg/dL	245 mg/dL	206.3	27 µU	63 µU	133.3	125 mg/dL	145 mg/dL	16.0	N/A	N/A	N/A	N/A	Otero-Losada, et al 2015	25
Wistar	HCHD[H]	30 sucrose	N/A	17	497.72	521.19	4.7	113.2 mg/dL	211.8 mg/dL	87.1	5.64 ng/mL	9.2 ng/mL	63.1	132.1 mg/dL	131.2 mg/dL	-0.7	6.9	12.0	74.7	Yes	Balderas-Villalobos, et al 2013	26
Sprague-dawley	HCHD[I]	66 sucrose	N/A	1	268	277	3.4	94 mg/dL	241 mg/dL	156.4	N/A	N/A	N/A	N/A	N/A	N/A	N/A	N/A	N/A	Yes	Reaven GM, et al, 1979	27
Sprague-dawley	HCHD[I]	66 sucrose	N/A	4	389	384	-1.3	84 mg/dL	270 mg/dL	221.4	N/A	N/A	N/A	N/A	N/A	N/A	N/A	N/A	N/A	Yes	Reaven GM, et al, 1979	27
Wistar	HCHD[H]	82 sucrose	N/A	39	700	800	14.3	1.28 mmol/L	1.69 mmol/L	32.0	6.61 ng/ml	6.79 ng/ml	2.7	9.78 mmol/L	9.23 mmol/L	-5.6	8.0	7.0	-12.5	No	Spadaro PA,et al ,2015	28
Wistar	HCHD[I]	66 fructose	5	16	454.6	390.7	-14.1	1.28 mmol/L	1.69 mmol/L	32	0.8 ng/L	2.4 ng/ml	200.0	N/A	N/A	N/A	8.2	11.2	35.9	Yes	Huang DW , et al, 2016	29
Wistar	HCHD[I]	65 fructose	5	2	180	163	-9.4	130 mg/g	45 mg/g	-65.4	N/A	N/A	N/A	N/A	N/A	N/A	8.2	8.2	0.0	N/A	Shimada M, et al , 2019	30
Sprague-dawley	HCHD[H]	60 fructose	8	8	320	200	-37.5	42.8	55.0	28.5	0.2	0.5	138.1	91.4	148.6	62.6	N/A	N/A	N/A	Yes	Shawky NM, et al, 2019	31
Wistar	HCHD[H]	32 sucrose	8	30	412	491.4	19.3	72.72 mg/dL	87.2 mg/dL	19.9	N/A	N/A	N/A	N/A	N/A	N/A	N/A	N/A	N/A	No	Durak A, et al , 2018	32
Wistar	HCHD[H]	33 sucrose +7 sucrose	4	4	254	273	7.5	N/A	N/A	N/A	N/A	N/A	N/A	N/A	N/A	N/A	1.4	3.0	114.3	N/A	De Queiroz KB, et al ,2014	33
Wistar	HCHD[H]	33 sucrose +7 sucrose	4	8	387.3	404.3	4.4	N/A	N/A	N/A	N/A	N/A	N/A	N/A	N/A	N/A	3.9	7.8	100.0	N/A	De Queiroz KB, et al ,2014	33
Wistar	HCHD[H]	30 sucrose	N/A	12	N/A	N/A	N/A	145 mg/dL	159.33 mg/dL	9.9	0.3	1.3	331.0	99.2 mg/dL	115.4 mg/dL	16.3	N/A	N/A	N/A	Yes	Olvera-Hernández V, et al , 2018	34
Wistar	HCHD[H]	30 sucrose	4	20	0	0	0	62.2 mg/dL	146.1 mg/dL	134.9	N/A	N/A	N/A	N/A	N/A	N/A	1.4	6.7	378.6	Yes	Acosta-Cota SJ, et al, 2019	35

Wistar	HCHD[H]	40 sucrose	4	20	420	820	95.2	62.2 mg/dL	93 mg/dL	49.5	N/A	N/A	N/A	N/A	N/A	N/A	1.4	6.2	342.9	Yes	Acosta-Cota SJ, et al, 2019	35
Wistar	HCHD[H]	50 sucrose	4	20	420	760	81.0	62.2 mg/dL	95.9 mg/dL	54.2	N/A	N/A	N/A	N/A	N/A	N/A	1.4	5.7	307.1	Yes	Acosta-Cota SJ, et al, 2019	35
Sprague-Dawley	HCHD[I]	65 sucrose	N/A	4	N/A	N/A	0.0	N/A	N/A	122.0	N/A	N/A	150.0	N/A	N/A	0.0	N/A	N/A	131	N/A	Chun MR, et al , 2010	36
Sprague-Dawley	HCHD[H]	30 sucrose	10	40	489.4	634	29.5	63 mg/dL	148 mg/dL	134.92	N/A	N/A	N/A	88 mg/dL	126 mg/dL	43.18	N/A	N/A	N/A	N/A	Espinosa-Juárez JV, et al , 2017	37
Sprague-Dawley	HCHD[H]	10 fructose	N/A	5	N/A	N/A	N/A	N/A	N/A	N/A	N/A	N/A	N/A	N/A	N/A	N/A	2.6	4.5	73.1	N/A	Li JX, et al ,2017	38
Wistar	HCHD[H]	29 starch + 29 sucrose	10	1.2	244	242	-0.8	0.85 mmol/L	1.56 mmol/L	83.5	N/A	N/A	N/A	N/A	N/A	N/A	N/A	N/A	N/A	Yes	Pôrto LC, et al , 2011	39
Wistar	HCHD[H]	25 fructose	8 to 10	16	422	446	5.7	N/A	N/A	N/A	N/A	N/A	N/A	N/A	N/A	N/A	119	356	199.2	N/A	Rickman C, et al , 2010	40
Wistar	HCHD[H]	68 sucrose	weaned	3.4	120	148	23.3	N/A	N/A	N/A	18.4 UI/mL	44.4 UI/mL	141.3	18.4mmol/L	44.4 mmol/L	141.3	N/A	N/A	N/A	Yes	Blázquez E and Lopez Quijada C, 1969	41
Harlan	HCHD[I]	87 fructose	9 to 11	12.8	476	424	-10.9	1.77 mmol/L	2.45 mmol/L	38.42	405 pmol	355 pmol	-12.3	8.26 mmol/L	9.63 mmol/L	16.59	N/A	N/A	N/A	N/A	Sharma N,et al, 2007	42
Wistar	HFD	45	8	15	402.73	391.24	-2.9	157.33 mg/dL	299 mg/dL	90.0	N/A	N/A	N/A	166.88 mg/dL	183.4 mg/dL	9.9	17.12	34.22	99.9	N/A	Ramalho L, et al, 2017	43
Sprague Dawley	LFD	20	8	8	100	125	25.0	1.3 mmol/L	0.95 mmol/L	-26.9	6.51 mU/L	10.47 mU/L	60.8	5.02 mmol/L	5.12 mmol/L	2.0	8	8	0.0	N/A	Cheng HS, et al, 2017	44
Wistar	HFD	40	N/A	12	315.87	370.04	17.1	98.05 mg/dl	125.23 mg/dl	27.7	1.33 ng/mL	2.89 ng/mL	117.3	5.84 mmol/l	10.63 mmol/l	82.0	1.31	1.71	30.5	N/A	Sour S, et al, 2015	45
Sprague Dawley	VHFD	60	4	10	291	346	18.9	0.36 mmol/L	0.543 mmol/L	50.8	4.38 μIU/mL	4.8 μIU/mL	9.6	6.05 mmol/L	6.7 mmol/L	10.7	2.85	3.09	8.4	Yes	Chung APYS, et al , 2014	46
Sprague Dawley	HFD	32.1	7	11	508	620	22.0	177 mg/dl	192 mg/dl	8.5	2.5 ng/ml	6.2 ng/ml	148.0	108 mg/dl	129 mg/dl	19.4	N/A	N/A	N/A	Yes	Fujimoto S, et al, 2010	47

Fischer	VHFD	68.48	4	13	290	320	10.3	N/A	N/A	N/A	1.06 ng/mL	2.08 ng/mL	96.2	6.2 mmol/L	6.9 mmol/L	11.3	1.9	2.7	42.1	Yes	Barbosa MA, et al, 2018	48
Wistar	HFD	50	N/A	20	245.55	342.86	39.6	N/A	N/A	N/A	16.33 µU/ml	31.46 µU/ml	92.7	83.59 mg/dL	241.39 mg/dL	188.8	N/A	N/A	N/A	N/A	Vinothiya K and Ashokkumar N, 2017	49
Sprague Dawley	HFD	45	4	15	520.17	713.09	37.1	1.14 mmol/l	1.95 mmol/l	71.1	N/A	N/A	45.9	7.69 mmol/l	11.22 mmol/l	45.9	6.33	11.15	76.1	N/A	Ha SK , et al, 2011	50
Wistar	VHFD	60	N/A	12	340	405	19.1	71 mg/dl	182 mg/dl	156.3	50 uU/ml	150 uU/ml	200.0	76 mg/dl	130 mg/dl	71.1	5.64	9.91	75.7	Yes	Sudhakara, G, et al, 2014	51
Sprague Dawley	LFD	29.5	N/A	21	362	527	45.6	105 mg/dl	280 mg/dl	166.7	2.2 l µU/ml	7.1 µU/ml	222.7	80.33 mg/dl	144 mg/dl	79.3	1.96	3.65	86.2	Yes	BrahmaNaidu P, et al, 2014	52
Sprague Dawley	HFD	50	6	8	184	294	59.8	66 mg/dl	158 mg/dl	139.4	1.2 ng/mL	4.2 ng/mL	250.0	113 mg/dl	147 mg/dl	30.1	0.9	1.7	88.9	Yes	Lee HJ, et al, 2017	53
Sprague Dawley	HFD	50	6	12	203	295	45.3	86 mg/dl	166 mg/dl	93.0	1.1 ng/mL	5.3 ng/mL	381.8	123 mg/dl	148 mg/dl	20.3	0.8	1.8	125.0	Yes	Lee HJ, et al, 2017	53
Wistar	HFD	42	6	12	504	606	20.2	1.2 mmol/l	2.1 mmol/l	75.0	577 pmol/l	780 pmol/l	35.2	5 mmol/l	5.4 mmol/l	8.0	N/A	N/A	N/A	Yes	Buettner R, et al, 2007	54
Wistar	LFD	10	N/A	7	135.46	156.92	15.8	0.661 mmol/L	1.724 mmol/L	160.8	N/A	N/A	N/A	N/A	N/A	N/A	3.75	6.08	62.1	N/A	Samout N,et al, 2016	55
Albino rats	HFD	58	N/A	8	N/A	N/A	N/A	N/A	N/A	N/A	11.12 µIU/mL	41.77 µIU/mL	275.6	75.25 mg/dL	112.65 mg/dL	49.7	N/A	N/A	N/A	Yes	Mehanna, et al, 2018	56
Wistar	HFD	40	8	8	425.4	508.5	19.5	64.4 mg/dl	74 mg/dl	14.9	N/A	N/A	N/A	108 mg/dL	128 mg/dL	18.5	16.4	48.46	195.5	N/A	Gomes DIN,et al, 2016	57
Wistar	HFD	59.5	7	12	373	551	47.7	43 mg/dL	72 mg/dL	67.4	N/A	N/A	N/A	105 mg/dL	187 mg/dL	78.1	7.5	13.6	81.3	N/A	Chien MY, et al, 2018	58
Wistar	HFD	45	6	8	327	388	18.7	0.9 mmol/L	1.35 mmol/L	50.0	23.23 mU/L	27.3 mU/L	17.5	5.72 mmol/L	9.84 mmol/L	72.0	N/A	N/A	N/A	Yes	Yang C, et al, 2019	59
Wistar	HFD	45	9	11	350	435	24.3	685.8 µmol/l	1366 µmol/l	99.2	66.66 IU/ml	84.65 IU/ml	27.0	N/A	N/A	N/A	8	14	75.0	N/A	Zhao L, et al, 2017	60
Wistar	VHFD	62.1	7	20	418.57	505.37	20.7	106.71 mg/dL	154.25 mg/dL	44.6	5.79 mU/mL	8.04 mU/mL	38.9	107.38 mg/dL	120.22 mg/dL	12.0	6.56	15.74	139.9	Yes	Kolahdouzi S, et al, 2019	61
Wistar	HFD	42	N/A	6	251.08	342.45	36.4	48.6 mg/ dl	109.105 mg/ dl	124.5	0.915 µg/ml	1.87 µg/ml	104.4	78.19 mg/dl	131.92 mg/dl	68.7	8.5	22.42	163.8	N/A	Haque MR and Ansari HS, 2018	62

Wistar	HFD	35	N/A	12	240.78	305.35	26.8	120.65 mg/dl	228.93 mg/dl	89.7	0.07 ng/ml	0.1 ng/ml	42.9	65.4 mg/dl	110 mg/dl	68.2	N/A	N/A	N/A	N/A	Ekeleme-Egedigwe A, et al, 2017	63
Sprague Dawley	HFD	40.8	N/A	18	500	635	27.0	0.43 mmol/L	0.76 mmol/L	76.7	15.29 uIU/ml	33.47 uIU/ml	118.9	5.13 mmol/L	5.32 mmol/L	3.7	18.04	43.74	142.5	Yes	Zhao M, et al, 2013	64
Wistar	HFD	59.28	N/A	12	424	545	28.5	N/A	N/A	N/A	1.93 ng/ml	3.66 ng/ml	89.6	147 mg/dl	149 mg/dl	1.4	N/A	N/A	N/A	Yes	Supakul L, et al., 2013	65
Wistar	HFD	59.28	N/A	16	461	567	23.0	N/A	N/A	N/A	1.97 ng/ml	3.84 ng/ml	94.9	155 mg/dl	161 mg/dl	3.9	27	63	133.3	Yes	Supakul et al., 2013	65
Sprague Dawley	HFD	40.8	7	18	490	610	24.5	N/A	N/A	N/A	194 pM	583 pM	200.5	5.04 mM	5.16 mM	2.4	9.59	20.35	112.2	Yes	Wang X, et al, 2013	66
Wistar	HFD	31	N/A	10	246.16	360.5	46.4	68.24 mg/dl	149.29 mg/dl	118.8	N/A	N/A	N/A	93.32 mg/dl	159.87 mg/dl	71.3	352.05	409.86	16.4	N/A	Kaur A, et al, 2019	67
Sprague Dawley	LFD	20	7	5	449.5	478.7	6.5	74.5 mg/dL	45.9 mg/dL	-38.4	N/A	N/A	N/A	N/A	N/A	N/A	18.1	23.4	29.3	N/A	Chiu CY, et al, 2017	68
Wistar	HFD	40	8	8	261.58	356.75	36.4	0.59 mmol/l	1.14 mmol/l	93.2	N/A	N/A	N/A	5.18 mmol/l	5.26 mmol/l	1.5	261.58	356.75	36.4	Yes	Zhang SJ, et al, 2011	69
Sprague Dawley	HFD	45	7	8	267.92	314.4	17.3	0.7 mmol/L	1.32 mmol/L	88.6	263.4 pmol/L	344.5 pmol/L	30.8	5.31 mmol/L	5.97 mmol/L	12.4	16.08	24.73	53.8	Yes	Li J ,et al, 2011	70
Wistar	HFD	45.7	14	2.5	393	430	9.4	N/A	N/A	N/A	137 pM	317 pM	131.4	4.3 mM	4.6 mM	7.0	N/A	N/A	N/A	Yes	Ciapaite J, et al, 2011	71
Wistar	HFD	45.7	14	25	505	655	29.7	N/A	N/A	N/A	200 pM	517 pM	158.5	4.9 mM	5 mM	2.0	N/A	N/A	N/A	Yes	Ciapaite J, et al, 2011	71
Sprague Dawley	HFD	42	N/A	8	300	325	8.3	82.8	152.3	84.0	9.29 µU/ml	27.19 µU/ml	192.7	64.33 mg/dl	104.03 mg/dl	61.7	9	15	66.7	Yes	Gomaa AA, et al, 2019	72
Sprague Dawley	LFD	10.1	N/A	15	363.8	458.5	26.0	93.1 mg/dL	166.7 mg/dL	79.1	3.2 µU/mL	7.02 µU/mL	119.4	79.3 mg/dL	135.75 mg/dL	71.2	N/A	N/A	N/A	Yes	Sakr HF, et al ,2017	73
Wistar	LFD	25	13	16	119.2	165.4	38.8	118 mg/dl	118.9 mg/dl	0.8	30.59 Pmol/L	51.75 Pmol/L	69.2	1265 mg/dl	2132 mg/dl	68.5	2.75	4.22	53.5	N/A	Farrokhfall K, et al, 2014	74
Wistar	VHFD	60	8	4	327.6	357.9	9.2	N/A	N/A	N/A	N/A	N/A	N/A	N/A	N/A	N/A	4.21	7.06	67.7	Yes	Osakabe N, et al, 2014	75
Wistar	VHFD	56	N/A	12	713.1	791.2	11.0	0.85 mmol/L	1.31 mmol/L	54.1	N/A	N/A	N/A	6.02 mmol/L	9.37 mmol/L	55.6	1.5	4.7	213.3	N/A	Wang Y, et al, 2012	76
Wistar	HFLCHD	CH= 37, L = 48	11	8	220.6	262.6	19.0	201.5 mg/dL	316.9 mg/dL	57.3	0.69 ng/mL	1.85 ng/mL	168.1	N/A	N/A	N/A	0.56	2.42	332.1	Yes	Ulla A, et al, 2017	77

Wistar	LFHCHD	CH= 51 , L= 35	8	10	390.9	483.6	19.0	64.1 mg/dL	104.4 mg/dL	62.9	29 µU/mL	59.3 µU/mL	104.5	74.3 mg/mL	80.8 mg/mL	8.7	N/A	N/A	N/A	Yes	Pons Z, et al , 2015	78
Wistar	LFHCHD	CH=82, L=18	3.5	8	312	383	22.8	0.89 mmol/L	1.4 mmol/L	57.3	0.5 ng/mL	1.4 ng/mL	180.0	3.5 mmol/L	4.9 mmol/L	40.0	N/A	N/A	N/A	Yes	Derkach KV,et al, 2017	79
Wistar	LFHCHD	CH= 70, L= 15	8	14	450	600	33.3	57 mg/dL	86.3 mg/dL	51.4	N/A	N/A	N/A	230 mg/dL	350.9 mg/dL	52.6	N/A	N/A	N/A	N/A	Parafati M, et al,2015	80
Wistar	LFHCHD	CH= 51, L= 33	9	4	373	449	20.4	1.91 mM	1.84 mM	-3.7	N/A	N/A	N/A	8.13 mM	9.2 mM	13.2	2.58	4.66	80.6	N/A	Sabater D, et al. 2014	81
Sprague-Dawly	LFHCHD	CH= 49 , L = 41	3	8	350	420	20.0	0.7 mmol/L	1.4 mmol/L	100.0	7.8 mmol/L	26.4 mmol/L	238.5	5.6 mmol/L	6.8 mmol/L	21.4	N/A	N/A	N/A	Yes	Lalanza JF, et al, 2014	82
Wistar	LFHCHD	CH= 35.2, L= 23.4	N/A	13	443	531	19.9	64.13 mg/dL	104.37 mg/dL	62.7	N/A	N/A	N/A	N/A	N/A	N/A	N/A	N/A	N/A	N/A	Baselga-Escudero L,et al, 2013	83
Wistar Kyoto	HFLCHD	L= 65%	10	4	307	328	6.8	75 mg/dL	200 mg/dL	166.7	35.9 µU/mL	56.9 µU/mL	58.5	117 mg/dL	151 mg/dL	29.1	N/A	N/A	N/A	Yes	Pasarín M, et al, 2012	84
Sprague dawley	LFHCHD	CH=51, L= 15	10	12	488	428	-12.3	N/A	N/A	N/A	3.7 ng/mL	3.4 ng/mL	-8.1	90 mg/dL	85 mg/dL	-5.4	N/A	N/A	N/A	No	Miesel A, et al, 2010	85
Sprague dawley	LFHCHD	CH=60.1, L= 25	10	12	470.4	529.6	12.6	N/A	N/A	N/A	2.3 ng/mL	3.6 ng/mL	56.5	83 mg/dL	93 mg/dL	12.0	N/A	N/A	N/A	Yes	Miesel A, et al, 2010	85
Wistar	HFLCHD	CH=66, L=24	8	16	577	500	-13.3	0.54 mmol/L	0.79 mmol/L	46.3	3.04 µg/L	4.15 µg/L	36.5	4.1 mmol/L	4.7 mmol/L	14.6	464 mg/mm	799 mg/mm	72.2	N/A	Panchal SK, et al, 2011	86
Wistar	LFHCHD	CH= 66, L=14	N/A	12	317	437	37.9	0.81 mmol/L	1.18 mmol/L	45.7	2.41 ng/mL	3.82 ng/mL	58.5	6.11 mmol/L	6.36 mmol/L	4.1	N/A	N/A	N/A	Yes	Bełtowski J, et al, 2009	87
Wistar	HFLCHD	CH= 31.5, L= 59.2	8	11	404.8	508.5	25.6	N/A	N/A	N/A	0.21 mg/L	0.38 mg/L	81.0	82.4mg/dL	87.1 mg/dL	5.7	60.65	94.91	56.5	N/A	García Díaz DF, et al,2007	88
Wistar	HFLCHD	CH= 31.5, L= 59.2	8	2	343.9	385	12.0	38.83 mg/dL	56.03 mg/dL	44.3	0.22 µg/L	0.36 µg/L	63.6	68.3 mg/dL	85.1 mg/dL	24.6	40.25	53.68	33.4	Yes	García-Diaz DF. Et al, 2007	88
Sprague Dawley	HFLCHD	CH= 27, L= 47	05-jun	8.6	443.78	540.2	21.7	2.27mmol/L	4.59mmol/L	102.2	19.75 Uu /Ml	98.04 Uu /Ml	396.4	12.8 mmol/L	12.1 mmol/L	-5.5	3.73	10.65	185.5	Yes	Darimon C, et al, 2004	89
Wistar	HFLCHD	CH= 27, L= 62	8	16	N/A	N/A	#¡VALOR!	1.4 mg/ml	1.09 mg/ml	-22.1	0.62 µg/l	1.83 µg/l	196.2	97.6 mg/dL	110 mg/dL	12.7	14.2	42.7	200.7	Yes	Reynes B, et al, 2001	90

Wistar	HFLCHD	CH= 27, L= 62	8	12	N/A	N/A	#¡VALOR!	1.68 mg/ml	1.31 mg/ml	-22.0	0.6 µg/l	1.48 µg/l	146.7	97.7 mg/dL	107 mg/dL	9.5	15.3	32.5	112.4	Yes	Reynes B, et al, 2001	90
Wistar	HFLCHD	CH= 33.5, L= 33.5	4	8	241	320	32.8	82.44 mg/dL	124.07 mg/dL	50.5	N/A	N/A	N/A	120.13 mg/dL	152.05 mg/dL	26.6	N/A	N/A	N/A	N/A	Bensalah M, et al, 2016	91
Wistar	HFLCHD	CH= 34, L= 64	60	14	12.8	129	907.8	N/A	N/A	N/A	68.3 µU/mL	261 µU/mL	282.1	8.01 mM	7.87 mM	-1.7	N/A	N/A	N/A	Yes	Gómez-Pérez Y, et al. 2008	92
Wistar	LFHCHD	CH=80, L=12	N/A	7	158.2	193.4	22.3	0.74 mmol/L	1.4 mmol/L	89.2	25.8 µU/mL	67 µU/mL	159.7	73.4 mg/dL	117 mg/dL	59.4	N/A	N/A	N/A	Yes	Pahua-Ramos ME, et al, 2014	93
Wistar	HFLCHD	CH=66, L=24	8	14	407.57	485.16	19.0	0.75 mmol/L	1.5 mmol/L	100.0	10.74 mmol/L	17.07 mmol/L	58.9	4.49 mmol/L	7.21 mmol/L	60.6	1.71	4.79	180.1	Yes	Hao L, et al, 2015	94
Wistar	LFHCHD	CH= 71, L= 22.9	8	20	80	120	50.0	38.76 mg/dL	57.97 mg/dL	49.6	4.15 µmol/mL	4.44 µmol/mL	7.0	226.4 mg/dL	327.7 mg/dL	44.7	3.65	6.1	67.1	No	Moreno-Fernández S, et al, 2017	95
Wistar	HFLCHD	CH= 41%, L= 47%	10	4	434	466	7.4	1.27 Mm	1.82 Mm	43.3	N/A	N/A	N/A	10.4 Mm	10.9 Mm	4.8	N/A	N/A	N/A	N/A	Oliva L, et al ,2017	96
Wistar	HFLCHD	CH=66, L=24	12	20	376.22	367	-2.5	0.35 mmol/L	2.44 mmol/L	597.1	N/A	N/A	N/A	N/A	N/A	N/A	6.8	15.2	123.5	Yes	Wong SK, et al, 2018	97
Sprague dawley	LFHCHD	CH=35 . L=61	7	4	489	529	8.2	N/A	N/A	N/A	186.1 pmol	286.9 pmol	54.2	6.14 mmol/L	6.27 mmol/L	2.1	N/A	N/A	N/A	N/A	Virtuoso et al 2018	98
Sprague dawley	LFHCHD	CH=35 . L=61	7	16	515	600	16.5	1.32 mmol/L	1.53mmol/L	15.5	227.7 pmol	289.2 pmol	27.0	5.2 mmol/L	6.01 mmol/L	14.4	N/A	N/A	N/A	N/A	Virtuoso A, et al, 2018	98
Wistar	LFHCHD	CH=66, L=24	8 to 10	4	110	110	0.0	65.3 mg/dL	67.3 mg/dL	3.1	N/A	N/A	N/A	64 mg/dL	64 mg/dL	0.0	N/A	N/A	N/A	N/A	Hazarika A, et al ,2016	99
Wistar	LFHCHD	CH=66, L=24	8 to 10	8	115	135	17.4	71.3 mg/dL	70.6 mg/dL	-1.0	N/A	N/A	N/A	64 mg/dL	61.4 mg/dL	-4.1	N/A	N/A	N/A	N/A	Hazarika A, et al ,2017	99
Wistar	LFHCHD	CH=66, L=24	8 to 10	12	150	180	20.0	64 mg/dL	118.6 mg/dL	85.3	N/A	N/A	49.3	67(mg/dL	100 mg/dL	49.3	N/A	N/A	N/A	No	Hazarika A, et al ,2018	99
Wistar	LFHCHD	CH=66, L=24	8 to 10	16	160	220	37.5	66 mg/dL	120 mg/dL	81.8	N/A	N/A	67.2	64 mg/dL	107 mg/dL	67.2	N/A	N/A	N/A	Yes	Hazarika A, et al ,2019	99
Wistar	LFHCHD	CH= 60, L= 25	8	6	341	425	24.6	49.54 mg/dL	57.88 mg/dL	16.8	N/A	N/A	N/A	67.92 mg/dL	71.4 mg/dL	5.1	N/A	N/A	N/A	N/A	Macedo IC, et al, 2012	100

Wistar	HFLCHD	N/A	3	14	421.57	464.93	10.3	98 mg/dL	105.16 mg/dL	7.3	1.09 ng/dL	1.18 ng/dL	8.3 8	99.1 mg/dL	105.62 mg/dL	6.6	0.92	1.57	70.7	No	Almeida FN, et al , 2013	101
Wistar	LFHCHD	CH= 65, L= 20	N/A	3	219	216	-1.4	42 mg/dL	114 mg/dL	171.4	32 µU/mL	50 µU/mL	56.3	116 mg/dL	113 mg/dL	-2.6	0.27	0.88	225.9	N/A	De Melo AF, et al, 2018	102
Sprague dawley	LFHCHD	CH= 60, L= 32	N/A	17	590	756.0	28.1	N/A	N/A	N/A	0.08 ng/mL	0.25 ng/mL	212.5	8.8 mmol/L	14.9 mmol/L	69.3	5.5	18.2	230.9	N/A	South T, et al, 2012	103
Sprague dawley	LFHCHD	CH= 50, L= 32	N/A	17	452.1	612.4	35.5	N/A	N/A	N/A	1.2 ng/mL	2.71 ng/mL	125.8	N/A	N/A	N/A	11.7	36.1	208.5	N/A	Manian J, et al, 2010	104
Sprague dawley	HFLCHD	CH= 44.5%, L= 43.4%	8 to 10	8	476.6	533.9	12.0	N/A	N/A	N/A	3 µU/L	2.8 µU/L	-6.7	13.1 mmol/L	12.4 mmol/L	-5.3	9.2	15.3	66.3	N/A	Ong ZY,et al, 2013	105
Sprague dawley	HFLCHD	CH= 38, L= 48	6 to 10	20	507.9	670.3	32.0	109.67 mg/dL	190.91 mg/dL	74.1	28.33 µIU/mL	95.5 µIU/mL	237.1	110 mg/dL	123 mg/dL	11.8	12.7	54.4	328.3	Yes	Lewis AR, et al, 2019	106
Wistar	HFLCHD	L=65	5	9.3	200.4	289.7	44.6	112.3 mg/dL	117.7 mg/dL	4.8	4.1 µU/Ml	4.5 µU/Ml	9.8	127.5 mg/dL	128.5 mg/dL	0.8	6	15.3	155.0	N/A	Lopes CO, et al, 2003	107
Wistar	LFHCHD	HC=63, L=29,	9 to 10	8	10.8	15.3	41.7	0.5 mmol/L	1.1 mmol/L	120.0	1 µmol/L	4 µmol/L	300.0	N/A	N/A	N/A	169.4	387.6	128.8	Yes	Poudyal H, et al, 2013	108
Wistar	HFLCHD	CH= 45, L= 37.8	8 to 10	12	221.66	295.16	33.2	73.71 mg/dL	200.58 mg/dL	172.1	30.55 µU/mL	50.34 µU/mL	64.8	80.68 mg/dL	104.14 mg/dL	29.1	N/A	N/A	N/A	Yes	Abd-Elwahab AH, et al, 2017	109
Wistar	HFLCHD	CH= 35, L=45-53	9 to 10	15	550	767	39.5	N/A	N/A	N/A	1.5 ng/mL	3.5 ng/mL	133.3	122.7 mg/dL	136.4 mg/dL	11.2	12	30	150.0	Yes	Sampey BP, et al, 2011	110
Wistar	HFLCHD	CH= 41.7, L=46.9	5	12	261.28	306.28	17.2	195.5 mg/dL	237.41 mg/dL	21.4	68.08 µU/mL	73.99 µU/mL	8.7	114.49 mg/dL	129.14 mg/dL	12.8	1.31	2.51	91.6	Yes	Buyukdere Y, et al, 2019	111
Wistar	HFLCHD	CH= 29 , L= 62	6	5	310.6	361.5	16.4	N/A	N/A	N/A	23.3 µU/mL	28.2 µU/mL	21.0	5.3 mg/dL	5.8 mg/dL	9.4	6.7	11.2	67.2	No	Pérez-Echarri N, et al, 2009	112
Wistar	HFLCHD	CH= 29, L=62	6	5	300	360	20.0	N/A	N/A	N/A	161.5 pM	195.8 pM	21.2	948 mg/L	1045 mg/L	10.2	6.7	11.2	67.2	No	Pérez-Echarri N, et al, 2009	112
Wistar	LFHCHD	CH= 65, L=16	N/A	16	513	603	17.5	0.72 mmol/L	1.19 mmol/L	65.3	31.4 µU/mL	49.5 µU/mL	57.6	4.82 mmol/L	5.82 mmol/L	20.7	29.8	49.5	66.1	Yes	Sishi B, et al,2011	113
Wistar	LFHCHD	CH= 35.2, L=23.4	8	16	475	652	37.3	N/A	N/A	N/A	3.23 µg/L	3.44 µg/L	6.5	5.18 mM	6.41 mM	N/A	8.0	17.5	120.1	Yes	Oliver P,et al , 2012	114

Wistar	HFLCHD	CH= 31.5, L=59.2	N/A	8	144	233	61.8	0.31 mm/dL	0.54 mm/dL	74.2	0.16 ng/mL	0.49 ng/mL	206.3	215 mg/dL	290 mg/dL	34.9	58.6	109.0	86.0	Yes	Milagro FI, et al, 2006	115

* The weight reported per week

$HCHD^{H}$ High Carbohydrate Diet Hypercaloric

$HCHD^{I}$ High Carbohydrate Diet Isocaloric

N/A does not apply
